# ‘Situation awareness’ in midwifery practice: a scoping review

**DOI:** 10.1136/bmjoq-2025-003724

**Published:** 2026-03-04

**Authors:** Rachael Budd, Paul Bowie

**Affiliations:** 1 School of Health Education Policing and Sciences, University of Staffordshire, Stoke-on-Trent, UK; 2 University of Glasgow, Glasgow, UK

**Keywords:** Human factors, Maternal Health Services, Patient safety, Systematic Review

## Abstract

**Background:**

Failure of situational awareness (SA) has been identified as a common theme in potentially avoidable maternal and infant deaths, although the empirical basis for this attribution is unclear. Situation awareness is arguably a contentious issue which needs to be studied methodically to ascertain the theoretical and practical relevance to midwifery to better inform the application of this concept to the clinical context—rather than seemingly and uncritically import the construct from other healthcare areas and safety-critical sectors unrelated to midwifery practice.

**Objectives:**

To identify how situation awareness is defined, understood, measured and interpreted within the midwifery care safety context as a precursor to further research which may contribute to improvements in safety of maternity care.

**Methods:**

A scoping review was conducted using a well-established methodological framework. A comprehensive literature search yielded 259 articles, of which 11 were included in the final review. Data from each article were extracted, charted and subjected to a thematic analysis.

**Findings:**

All primary research papers applied Endsley’s original definition of situation awareness, either explicitly or implicitly. Team SA was viewed as an aggregate of individual clinicians’ SA. Only two of the studies attempted to measure SA; others made inferences about levels of SA based on observable features of teamwork.

**Conclusions:**

Endsley’s model of SA has been applied to midwifery without full consideration of whether this theoretical construct is appropriate for this clinical context. Other extended SA models exist which could arguably provide a more informed systems-theoretic approach to maternity care safety, consistent with the current drive towards embedding systems thinking and creating a Just Culture in healthcare organisations.

**WHAT IS ALREADY KNOWN ON THIS TOPIC**

- The quality and safety of maternity services has been a focus of international policy influenced by related research and independent safety investigations.
- Adverse outcomes in midwifery care have frequently been linked to ‘loss of situation awareness’ in successive high-profile safety reports.

**WHAT THIS STUDY ADDS**

- Situation awareness is overwhelmingly viewed as a cognitive state in the midwifery literature.
- The cognitive perspective is controversial and potentially limited because it focuses solely on the mental processes of individual practitioners and may also be associated with blame if an adverse outcome occurs.
- Other theoretical models of situational awareness exist, which consider the influence of broader sociotechnical system elements on performance outcomes.

**HOW THIS STUDY MIGHT AFFECT RESEARCH, PRACTICE OR POLICY**

- Further research is needed to investigate whether an alternative model of situation awareness could be applied to midwifery to explain systems factors which affect the performance of midwives generally, and more specifically their ability to recognise patient deterioration (where ‘loss of situation awareness’ is frequently cited as a contributory factor).

## Introduction

### Maternity safety

 While childbirth is deemed to be relatively safe in the UK and most modern health systems,^1^ mortality has not been eradicated. Currently, there are 5.8 stillbirths or neonatal deaths per 1000 births and 7 maternal deaths per 100 000 maternities in the UK^1^ out of 716 000 annual births.^2^ By contrast, countries such as Norway and Sweden have achieved mortality rates as low as 3.8 and 3.9 stillbirths and neonatal deaths per 1000 births respectively, and 2 or 4 maternal deaths per 100 000 births, respectively.^1^

The UK Government has set an ambition to reduce the rate of stillbirths, neonatal and maternal deaths in England by 50% by 2025.^3^ Therefore, it is of paramount importance to identify factors which contribute to maternal and infant deaths to improve the safety of maternity care. Concerningly, the most recent maternal mortality report identified that in 37% of cases of maternal death, improvements in care may have changed the outcome.^4^ Maternity cases account for around 60% of the £9 billion that National Health Service (NHS) Resolution spends annually on clinical negligence claims.^5^ This figure is not only unsustainable but also takes funding away from direct care provision where it is needed.^6^ Clearly, work is required to improve safety in maternity services to reduce patient harm and consequentially minimise the financial burden of negligence claims to the NHS.^6^

### ‘Situation awareness’ in maternity services

Successive reports into the safety of maternity services have cited failure to recognise patient deterioration due to ‘loss of situation awareness’ as a contributory factor to potentially avoidable maternal and infant deaths,^27^,^10^ although the empirical basis for this attribution is unclear. The relevance of situation or situational awareness (SA) to safety performance is a well-known construct in industries such as aviation, nuclear energy, the military and the oil and gas sectors.^11^ While it has been recommended that ‘human factors’ lessons be learnt from other high-risk industries, to minimise safety occurrences and thus improve patient safety,^12^ it is argued that concepts should not be indiscriminately transferred from one context to another.^13^ Within the Human Factors professional community, SA as a construct is a contentious issue, which attracts heated debate that appears to be largely unknown about in healthcare. Some authors have questioned whether SA actually exists as an entity and significant debate exists as to whether SA is an operational or representational concept.^14 15^ Consequently, SA needs to be studied methodologically to ascertain the theoretical and practical relevance to midwifery to improve understanding of this issue and tailor the application of this concept to the clinical context.^13 16^

Against this background, the objective of this review is to identify how SA is defined, understood, measured and interpreted within the midwifery care safety context as a precursor to further research on how understanding of SA may be enhanced to monitor and improve care performance and consequently the safety of maternity care.

## Methods

### Scoping review

Munn *et al*^17^ state that scoping reviews can be undertaken to map existing literature on a topic to understand how a concept is defined and to identify gaps in the literature. The present review meets all of these criteria. A scoping review has been conducted using Arksey and O’Malley’s^18^ methodological framework which consists of five stages:

1. Identifying research questions.
2. Identifying relevant studies.
3. Study selection.
4. Charting the data.
5. Collating, summarising and reporting the results.

These stages have been used to structure the report which follows.

### Stage 1: identifying the research question

The intrapartum period is a time of great physiological change, with the potential for rapid deterioration in health of the mother and baby. SA is thought to be important in this context because the role of the midwife is to detect deviations from the normal physiological course of labour and act promptly to access emergency care where necessary.^19^ Therefore, the concept in this scoping review is situation awareness and the context is intrapartum care. Within the literature, the terms situational and situation awareness are used interchangeably, with more recent work tending to use the latter.^20^ Both terms were used in the search strategy to ensure comprehensive retrieval of literature; however, only ‘situation awareness’ will be used in this article, for consistency.

The participants in this scoping review are midwives because this professional group is the main care givers for women in labour, with one-to-one midwifery care being recommended by the National Institute for Health and Care Excellence.^21^ In summary, the research question posed is: How is ‘situational awareness’ defined, understood, measured and interpreted in research related to intrapartum care by midwives?

### Stage 2: identifying relevant studies

The literature search adopted a variety of strategies to identify relevant articles. First, CINAHL, Scopus, Psych Info and PubMed databases and the University’s library search engine were searched using the key terms situation awareness or SA and combined with the Boolean operator AND Midwifery or Midwives or Midwife or Maternity. It was not necessary to limit the search by year of publication as each search retrieved a manageable number of publications to hand sift for relevance; however, results were limited to English language for practical reasons.

Reference lists of retrieved papers were searched for additional relevant papers and key journals such as the Journal of Interprofessional Care, Human Factors, Applied Ergonomics, Ergonomics, Safety Science and the British Journal of Midwifery were hand-searched. Finally, grey literature was searched via the British Library’s Ethos service for PhD theses, websites of relevant organisations such as the Royal College of Midwives (RCM), the RCM i-learn portal, the Royal College of Obstetricians and Gynaecologists (RCOG), the Healthcare Safety Investigation Branch (HSIB), NHS Improvement and The Healthcare Improvement Studies Institute and the Chartered Institute of Ergonomics and Human Factors. Although the National Patient Safety Agency has closed, the website materials are archived online, and this was searched for historical literature.

### Stage 3: study selection

**Figure 1:**
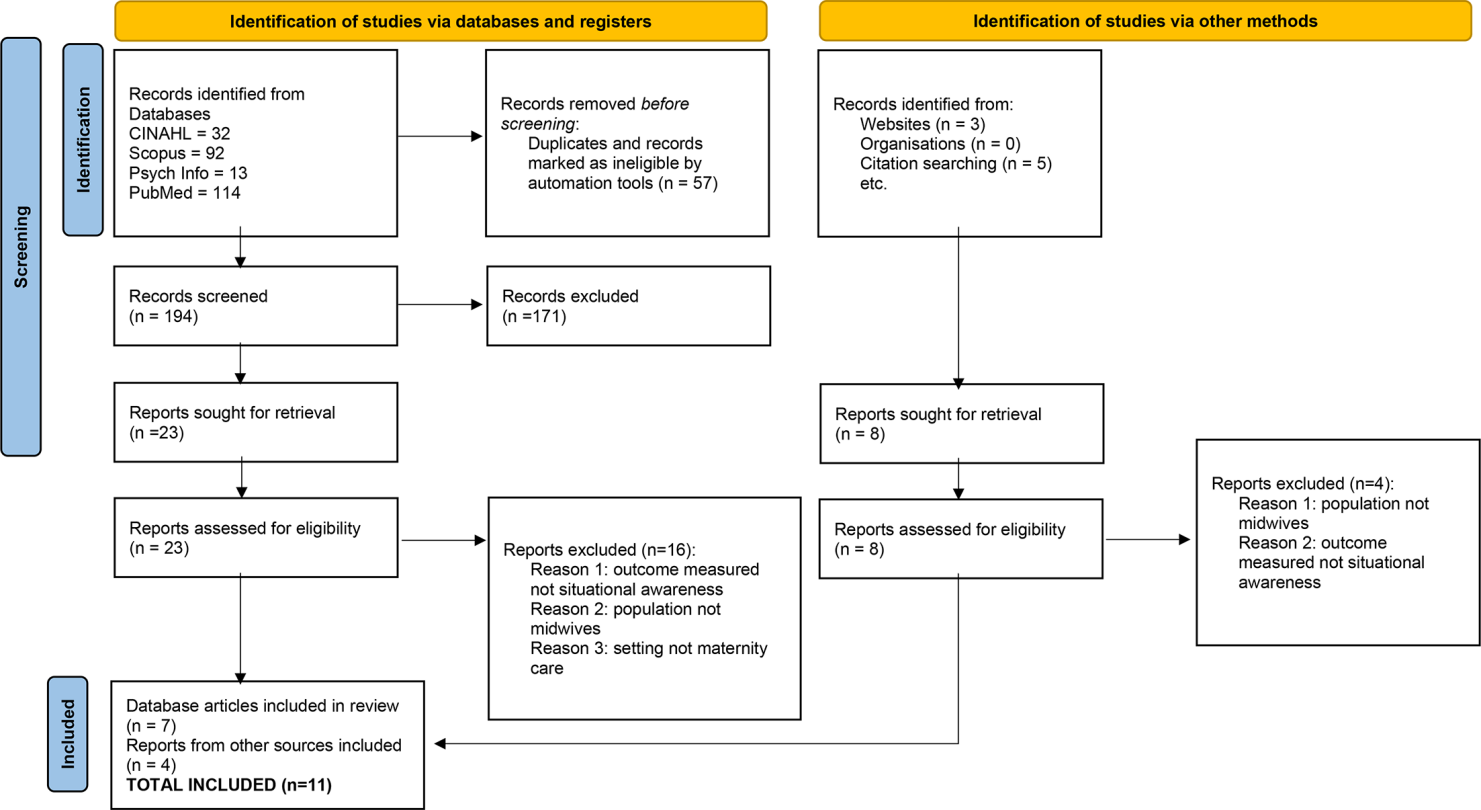
PRISMA flow diagram for article selection. PRISMA, Preferred Reporting Items for Systematic Reviews and Meta-Analyses.

Using the key search terms identified above, 259 articles were retrieved. Duplicates were removed and then the title and abstract of all the remaining articles were screened by the author for eligibility. As with Arksey and O’Malley’s^18^ method, inclusion and exclusion criteria were applied post hoc, once the breadth of the field had been surveyed. The criteria used can be seen in table 1, while the Preferred Reporting Items for Systematic Reviews and Meta-Analyses diagram to illustrate how the final selection of papers was chosen is detailed in figure 1.

**Table 1:** Inclusion and exclusion criteria

Criteria	Inclusion	Exclusion	Rationale
Study focus	Situation awareness	Situation awareness mentioned but not studied	Some studies mention SA within the background, but this is not the topic under investigation.
	Non-technical skills where SA included SA	Non-technical skills where SA not included	‘Non-technical skills’ covers a range of skills which may or may not include SA.
		Teamwork	Teamwork and communication skills are related to SA but are separate concepts.
		Communication skills	
Context	Labour ward	Operating theatres	Care not provided by midwives in these settings and patients are not necessarily pregnant/intrapartum.
	Intrapartum care	Emergency department	
	Maternal or fetal care	Neonatal care	Neonatal care generally pertains to care on a neonatal intensive care unit by neonatal nurses, not midwives.
	Simulated intrapartum care		It may not be ethical to conduct research with women in labour.
Participants	Midwives/Midwife/Midwifery	Obstetrics (relating to doctors only)	SA of obstetricians and anaesthetists may be different to that of midwives. The focus of this scoping review is midwives.
		Anaesthetic	
	Multidisciplinary obstetric where midwives were included in this population	Multidisciplinary teams that did not include midwives	Midwives work as part of the multidisciplinary team. Multidisciplinary populations were included so long as they included midwives.
Geographical location	UK or other modern international healthcare systems	Low- or middle-income countries	Healthcare context likely to affect understanding of SA considering disparity between maternal mortality and morbidity rates in high- and low- to middle-income countries.^48^

SA, situational awareness.

### Stage 4: charting the data

The key features of each of the research studies were charted in online supplemental material appendix 1, including the definition of SA and measurement tool used in each article.

### Stage 5: collating, summarising and reporting the results

To depict the breadth and nature of the literature, a numerical overview of the included articles is provided in table 2. Clearly, the heterogeneity of the methodologies means that it is not possible to synthesise the results, nor was it desirable to do so in this scoping review. The objective was to identify how SA was defined in the literature, and how it has been measured. The findings are presented thematically under those headings. Team situation awareness (TSA) was an aspect of the definitions of SA which arose from the literature and warranted consideration under its own heading as there was significant variation in how team SA was defined and understood.

**Table 2:** Characteristics of included articles (n=11)

Year of publication	2005–2009	1
	2010–2014	2
	2015–2019	6
	2020>	2
Geographical location	UK	8
	Australia	1
	Canada	1
	USA	1
Type of publication	Research	6
	Commentary	2
	Patient safety report	2
	e-learning package	1
Research methods (not mutually exclusive)	Observation	2
	Simulation	2
	Programme evaluation/feasibility	2
	Document analysis	1
Populations	Multidisciplinary team (MDT)	4
	Labour ward coordinators	1
	Student midwives	1

## Findings

### Definitions of situation awareness

The grey literature and four out of the six primary research studies in this scoping review defined SA using Endsley’s model (box 1). One study^22^ cited Wright *et al*^23^ in their definition, although this article is based on the original work by Endsley, who is also co-author of the paper. Wright *et al* very similarly define SA as ‘a person’s perception of elements in the environment, comprehension of that information, and the ability to project future events based on this understanding’. Despite the title of Bunford and Hamilton’s^24^ paper containing the word SA, the term is not actually defined within the paper; however, Wright *et al*’s (2004) article is mentioned within the discussion section. Consequently, it can be said that all of the primary research papers applied Endsley’s (1995) definition of SA,^25^ either explicitly or implicitly.

Box 1 **Endsley’s definition of situation awareness**

The perception of the elements in the environment within a volume of space and time, the comprehension of their meaning and the projection of their status is the near future (Endsley, 1995: 36).

In all but one of the papers included, SA was viewed as a person-level cognitive concept, whereby SA is assumed to be held solely within the mind of the individual. However, there was division between the definitions in the theoretical commentaries as to whether SA is a cognitive state^26^ or a cognitive process.^27^ A state infers possession of SA as an entity whereas a process describes the action of obtaining that awareness. This is an important distinction because of the implications it may have for how SA should be measured, by process or outcome measures, if indeed cognitive functions can be measured.^28^ Endsley, who is cited in both commentaries, argues that SA is a cognitive state,^25^ choosing instead to define the process of acquiring SA as ‘situation assessment’. The HSIB cites Endsley’s 1988 definition of SA,^10^ which, as stated above, views SA as a cognitive concept; however, the report considers the construct from a systems perspective, recommending that ‘situation awareness is more appropriately seen as the outcome of the interaction between staff and all the other elements that make up a work system and hence is an organisational issue’.^10^ This is the only publication within this scoping review to consider SA as a systemic construct, although the theoretical model they have cited does not appear to fully align with this view.

### Team situation awareness

**Figure 2:**
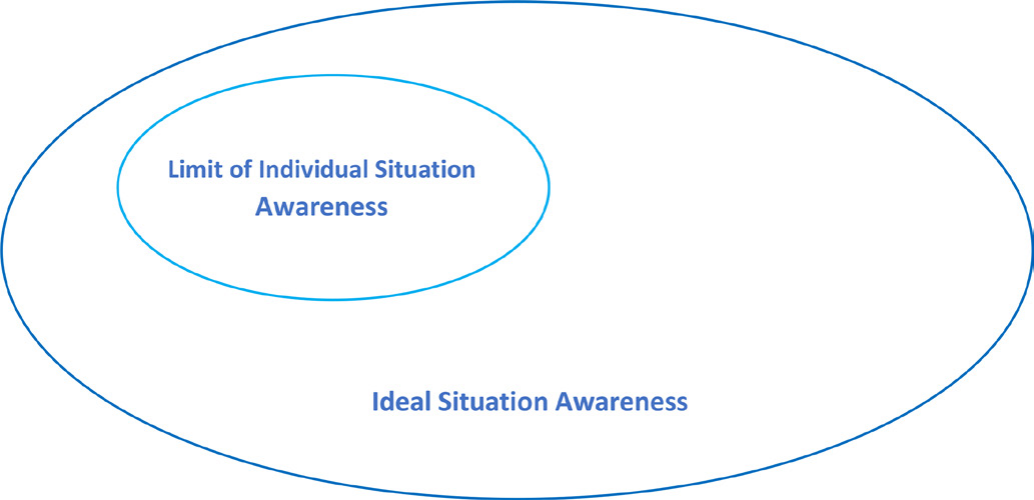
Model of ideal and practical situational awareness. Reproduced from Royal College of Obstetricians and Gynaecologists (2017, p. 61) with the permission of the Royal College of Obstetricians and Gynaecologists.

The concepts of individual and TSA are intertwined in Rayfield *et al*’s definition,^27^ in which ‘individuals and teams gain complete awareness and understanding of the situation around them…. and requires individuals to work together in order to share knowledge, to perceive the elements in their environment, to develop understanding of their meaning and to make projections and plans in the near future’.^27^ The authors go on to say that sharing individual perception and comprehension of a situation leads to ‘ideal situational awareness’^27^, although the meaning of this term is not elaborated on. The RCOG^9^ also presents the same graphic in their report (figure 2) which depicts the limits of individual situation awareness within the bigger area of ideal situation awareness. They also suggest that individuals generate their own SA which can then be communicated with others. These definitions appear to infer that TSA is built as a cumulation of individual perceptions and comprehension.

Team SA was also considered to be an aggregate of individual clinicians’ SA within two of the primary research papers.^22 29^ Meanwhile, Morgan *et al*^30^ state that team members can share SA but do not elaborate on how this occurs; it is not clear whether this is a cumulation of individual SA or some other mechanism, for instance, dissemination of one person’s expertise. On the contrary, the study by Sonesh *et al*^31^ which evaluates a team training programme does not mention team SA at all, with teamwork discussed as a separate issue to SA.

### Measurement of SA

Only two out of the six primary research studies attempted to measure SA. Morgan *et al*^30^ used the Situation Awareness Global Assessment Technique (SAGAT) developed by Endsley to measure SA in their interprofessional training programme.^32^ It is clear from the thorough explanation of their methods that the authors Morgan *et al*^30^ have faithfully adhered to the theoretical foundations in developing their application of the technique. Cooper *et al* state within the introduction to their paper that the SAGAT tool can be used to measure SA in simulated situations;^33^ however, it is not clear whether this is the method that they actually employed as SAGAT is not mentioned again within the rest of the paper. There are some similarities between Cooper *et al*’s method^33^ and the SAGAT tool devised by Endsley,^32^ namely the use of Goal Directed Task Analysis in the development of the scenarios and questions to be asked of participants. In keeping with Endsley’s method^32^ and the approach taken by Morgan *et al*,^30^ Cooper *et al*^33^ also asked participants questions about their knowledge of the situation which was compared against the correct answer to provide an SA score. However, there are also stark differences between the methods such as administering all of the questions after the completion of the simulation rather than freezing the situation at random points throughout as Endsley recommends.^32^ Thus, it is not clear whether Cooper *et al*^33^ had intended to use a modified version of the SAGAT or had devised their own method entirely.

Contrary to the studies above which attempted to quantitatively measure SA, Abbott *et al*^22^ report various behaviours which they say affected levels of TSA; however, TSA awareness was not actually measured. In this study, observable features of teamwork such as cooperation, co-ordination, leadership, monitoring and communication were narratively equated with various levels of TSA. For example, ‘the Red Delivery Unit team seemed less integrated and more fragmented, and as a result the exchange of information was less habitual and less effective, leading to lower TSA’.^22^ It is not clear how the researchers assessed that levels of TSA were lower at this study site.

Similarly, Mackintosh *et al*^29^ aimed to describe the main mechanisms supporting TSA, suggesting how mechanisms such as the White Board, handover and the role of the Delivery Suite Coordinator affected levels of TSA. However, as a qualitative study, TSA is not measured in any way, so these connections are theoretical. The findings sound credible in terms of how information is shared, but the link between these mechanisms and cognitive processes which define TSA is not adequately considered.

Finally, SA was included as part of Sonesh *et al*’s training programme;^31^ however, SA itself was not measured as an outcome. Instead, knowledge of SA was measured within the pre- and post- training knowledge tests, although the findings suggest that the training did not significantly improve knowledge of SA. Clearly, improvement in knowledge may be a useful measure of the effectiveness of a training programme; however, the usefulness of knowing about SA is questionable.

## Discussion

This scoping review sought to map the breadth of existing literature and identify how situation awareness is defined, applied and measured in the midwifery literature. A comprehensive search of the academic and grey literature found only six primary research papers and five other non-empirical publications. There was a breadth of coverage in terms of the heterogenous aims and methodologies of the research and geographical location of the studies; however, definitions of SA were largely ubiquitous.

### SA as a cognitive construct dominates

The most important finding of this review is that all of the existing midwifery literature on SA is based on Endsley’s original theory.^32^ This finding is in keeping with the wider body of literature in which Walshe *et al* found that 80% of papers included in their meta-narrative review of nursing practice applied Endsley’s model.^34^ Endsley’s model has been criticised because it focuses on the cognitive processes within the mind of the individual clinician and does not fully account for the contribution of interacting systemic factors such as the physical, technological, social, policy and regulatory environments to the decisions that are being made,^35 36^ as well as the influence on performance of the complexity of everyday care delivery (eg, patient complications, unnecessary distractions, the need to manage goal-conflicts and make efficiency-thoroughness trade-offs).^37^ Furthermore, viewing SA as a purely cognitive construct may inadvertently lead to blame being apportioned to the individual(s) if an adverse outcome occurs.^15^ In contemporary healthcare, there has been a move towards establishing a ‘system approach’ which incorporates both the organisational need to build a fair and open Just Culture to inform learning from patient safety incidents and also exploring the dynamic interacting contributory factors that inevitably influence how these incidents arise. This is in contrast to the common approach in healthcare which involves attempts to go ‘down and in’ to identify ‘root causes’ with a tendency to also overly focus on the (in)decisions and (in)actions of people directly involved. It is worth noting that the application of linear and reductionist-type root cause analysis approaches to complex healthcare problems is beginning to be called into question.^38^,^40^ This can sometimes result in punitive consequences for some and frequent missed opportunities to learn more meaningfully for the teams and organisations involved. Indeed, Walshe *et al* conclude that there is a need for further research which moves away from the overriding cognitive behavioural tradition to reflect on the realities of nursing work within highly complex socio-technical teams, where the ability to flex and adapt to changing organisational conditions is often key to success.^34^ This is supported by the HSIB report^10^ which recommends that SA should be viewed as an organisational construct, rather than as an individual behavioural state.

### Models of situation awareness

Within the literature, team SA was considered to be a cumulation of individual SA. The diagram presented by Rayfield *et al*^27^ and the RCOG^9^ (figure 2) infers that by sharing information, all members of the team will subsequently have the same understanding of the situation, generating a much larger area of team SA which they label ‘ideal SA’. While Wright and Endsley^41^ do state that SA can be shared within a system through TSA, they draw an important distinction between SA which is complementary and that which is shared. The assumption that all members of the team will have the same understanding of the situation does not take account of the nuanced roles and perspectives of different professionals within the team. Although clinicians may have access to the same information, it is unlikely that they will construct the same mental model of the situation given their bespoke perspectives as a result of differing past experience, personal beliefs and biases.^41^ This theoretical debate brings into question the validity of measuring team SA by aggregating individual scores. Measurement of SA is notoriously problematic because cognitive processes cannot be directly observed.^42^ This may explain why only two of the studies in this review sought to try to measure it. Interestingly, Morgan *et al* concluded from their research that it may not be necessary for all team members to have all of the information; it may be sufficient for individuals to have only that information which is required for their own role within the situation.^30^

An alternative model of SA exists, which has not been considered within the midwifery context, but which aligns more closely with Morgan *et al*’s conclusion.^30^ Distributed situation awareness (DSA) advocates that within collaborative environments, there exists a collective awareness which consists of ‘each element’s compatible portion of SA’.^43^ That is, the individual holds their own awareness, which is necessary for their own role and tasks. While information may be shared between elements of the system, the SA itself is not shared because of the idiosyncratic construction of SA, based on individuals’ different knowledge, experience, personal biases, etc. Thus, SA is distributed across the team. Research has suggested that DSA may be a more appropriate model to use within highly complex socio-technical systems,^44^ and it has been found to effectively model SA in patient flow.^45^ Therefore, this type of systemic-theoretical model may have greater potential utility in Midwifery practice than the models included in this review.

### Strengths and limitations

Arksey and O’Malley’s framework^18^ enabled a systematic and structured approach, which has been transparently reported, thus could be reproduced within other allied fields of healthcare. The scoping review method was chosen for its ability to map a diverse range of literature, such as the articles included within this review. Although the intention was not to synthesise the result, the heterogeneity of the identified studies precludes the ability to draw reliable conclusions from the existing literature. However, scoping the operationalisation of SA has enabled comparisons to be made with the theoretical foundations of the concept. This has revealed opportunities for further work, taking a systems perspective conducive with the current move towards a Just Culture. A weakness of this review is that the literature search and selection of studies was conducted by only one researcher; therefore, there is potential that some research may have been missed.

### Study implications

There is arguably a clear learning need within maternity policy, research, education and practice around the understanding and application of the concept of situation awareness to inform both learning and conclusions arising from unwanted safety occurrences. Dekker outlines four consequential issues that should be addressed by the aforementioned stakeholders^15^:

1. That SA should not be linked as a measure of professional conduct and that rather it is a system outcome and not a person-level ‘direct action or virtue’.
2. The frequently cited term and assumed outcome of ‘loss of situation awareness’, especially as part of maternity safety investigations, leads to the assignment and shifting of (either directly or indirectly) from the organisational level to the person level.
3. As this study has found, Dekker also points to the sheer difficulty in adequately defining and accurately measuring SA given that it (whatever ‘it’ is) is heavily influenced by interacting and sometimes unpredictable systemic factors in highly complex adaptive organisations where, arguably, most outcomes (including SA) are emergent properties of these systems.^46 47^
4. There is a need to move beyond the psychological perspective of SA as a person-level cognitive construct, with team SA considered as a summation or aggregate of individuals’ SA. The field of human factors and ergonomics, with its foundational ‘systems approach’ to problem analysis and work system redesigns, recognises the controversial debate around these issues (largely unknown or acknowledged in healthcare circles) by arguing that to better understand the concept of SA more comprehensively, then the unit of analysis should be the whole sociotechnical work system in which people inhabit and function.^36^

## Conclusion

Within the midwifery literature, SA is universally viewed from a behavioural psychological perspective as a person-level cognitive construct. This view is potentially problematic when it is applied to complex socio-technical systems, because it may not always account for the interaction between elements of the broader care system and their influence on the behaviour and performance of the individual midwife. There are also conceptual issues with the notion of team SA, as a cumulation of individual SA, which fails to take account of the nuanced roles and expertise of individuals within the interprofessional team.

While thus far only Endsley’s 1988 model^32^ has been considered in midwifery, Stanton *et al*^20^ advocate that there is not one omnipotent model of SA, but that an appropriate model should be chosen based on the context. Consequently, the findings of this scoping review present an opportunity for further research to investigate whether an alternative model of SA may be more appropriate in the midwifery context, reflecting the complex socio-technical system in which midwives typically work. Although not previously applied to midwifery, DSA may be a more appropriate model to use within this type of complex socio-technical system.^44^ ^47^ Further research is needed to consider whether the DSA model could be applied to midwifery with practical utility for improving system awareness and thus improving the safety performance of maternity care.

## Supplementary material

10.1136/bmjoq-2025-003724 online supplemental file 1

## Data Availability

Data sharing is not applicable as no datasets were generated and/or analysed for this study.
