# Supplementary material for: ‘Situation awareness’ in midwifery practice: a scoping review
Source: BMJ Open Qual. 2026 Mar 4;15(1):e003724. doi: 10.1136/bmjoq-2025-003724 (PMC12970059; doi:10.1136/bmjoq-2025-003724)
Supplement: online supplemental file 1 [file bmjoq-15-1-s001.docx]

**Appendix 1 (Supplementary material): Scoping Review Results Chart**

| **Reference and location** | **Population** | **Aim** | **Methodology** | **Intervention** | **Definition of SA** | **Measurement of SA** | **Outcome measures** |
| --- | --- | --- | --- | --- | --- | --- | --- |
| **Abbott, S., Rogers, M. and Freeth, D. (2012)**  UK – one inner city London hospital, one provincial city hospital. | Multidisciplinary team on labour wards at 2 hospitals. | To examine how customary team behaviours contribute to safety culture | Non-participant Observation  Part of a bigger study which included clinical audit. | N/a | Wright et al (Wright, 2004): perception and understanding of environmental events and consequences and to plan accordingly.  Team SA (TSA) is built from team members’ individual SA and mental models. | Not measured. | Observable features of teamwork as proxies of SA e.g. cooperation, co-ordination, leadership, monitoring and communication. |
| **Bunford, D. and Hamilton, S. (2019)**  UK (national) | Labour ward co-ordinators | To analyse job descriptions to gain an understanding  of organisations’ expectations of the delivery suite  co-ordinator role. | Qualitative documentary analysis of 15 Labour ward co-ordinator job descriptions. | n/a | Wright et al (Wright, 2004) 3 levels: ability to perceive elements of the environment, comprehension of the situation and ability to project into the future situation* | Not measured. |  |
| **Cooper, S., Bulle, B., Biro, M.A. et al. (2012)**  Australia | Student Midwives | To examine the ability of student midwives to assess and manage maternal deterioration in a simulated setting. | Exploratory quantitative analysis of student performance | 2 x 8 minute simulation exercises, simulating APH and PPH scenarios. | Endsley’s model | 15 yes/no questions developed using Goal Directed Task Analysis. Scores were calculated from the number of correctly answered questions. | Knowledge.  Situation awareness.  Skill performance |
| **MacKintosh, N., Berridge, E.-. and Freeth, D. (2009)**  UK. 4 Delivery suites: 2 northern hospitals, 2 London borough hospitals | Delivery Suite co-ordinator Midwife, Midwives, obstetricians | To describe the main mechanism supporting team situational awareness (TSA) and examine contrasting configurations of supports. | Lightly structured ethnographic observation.  Refinement of themes by focus group discussion | n/a | Endsley’s model of individual SA.  TSA built of individual situation models and shared mental models. | Not measured. | Sensitising concepts: safety culture, non-technical skills, teamwork and decision making. |
| **Morgan, P., Tregunno, D., Brydges, R. et al. (2015)**  Canada | Three teams of 1 obstetrician, 1 anaesthesiologist and  3 registered nurses | pilot study to evaluate validity of using the SAGAT tool in three interprofessional team simulation training scenarios. | Feasibility study comprised of questionnaires of participant opinion and SAGAT tool to assess performance in simulated emergencies. | Three simulations of clinical emergencies; | Endsley’s model | SAGAT | Individual participant SAGAT score  Team scores: cumulative score from the proportion of correct responses by all members of the team |
| **Sonesh, S.C., Gregory, M.E., Hughes, A.M., et al. (2015)**  USA | Nurses and physicians | To assess the effectiveness of a team training intervention in improving learning and transfer of teamwork, SA, decision making, and cognitive bias as well as patient outcomes in Obstetrics. | Repeated measures multilevel evaluation | STEPPS Programme | Endsley’s model | SA included as part of the training but SA itself not measured as an outcome. Knowledge of SA was measured within the pre- and post- training knowledge tests. | Participants’ satisfaction with the training.  Skills transfer.  Perceptions of teamwork transfer.  decision making accuracy.  clinical outcomes. |
| **Edozien, L.C. (2015)**  UK | Predominantly aimed at obstetricians but does mention Midwives and multidisciplinary team | To provide an overview of SA as a nontechnical skill that is essential  for safe practice in a delivery suite. | Commentary | n/a | Endsley’s model | n/a | n/a |
| **HSIB (2020)** | n/a | To explore the patient safety risk of delays to intrapartum intervention once fetal compromise has been identified,  through an organisational resilience lens. | National investigation, included review of HSIB maternity investigations, plus findings of national reports, observation and interviews at two maternity units, and expert opinion. | n/a | An awareness and understanding by staff of everything that is going on around them and its potential effects.  Cites Endsley’s definition (Endsley, 1988). | Not measured | n/a |
| **Rayfield, M., Ansari, S. and Prosser-Snelling, E. (2017)**  UK | n/a | To highlight the contribution of a loss of situational awareness to the mortality and morbidity of mothers and babies, explain the meaning of situational awareness and explore its barriers and facilitators | Commentary | n/a | Ensdley’s model |  | n/a |
| **Rayfield, M. (2021)**  UK | n/a | To give midwives an understanding of human factors and how these apply to their role and to  provide material to help reduce human factor errors in midwifery | Human Factors e-learning training package | n/a | “The cognitive process through which we can gain an awareness of the whole situation: the helicopter view”. |  | n/a |
| **RCOG (2017)** | n/a | to share the lessons from the care of ‘Each Baby Counts’ babies born  in 2015 | National quality improvement programme report, reporting on data relating to the care of 1136 babies born during 2015 that met the inclusion criteria. | n/a | Endsley’s definition | n/a | n/a |
